# The Relationship between Social and Motor Cognition in Primary School Age-Children

**DOI:** 10.3389/fpsyg.2016.00228

**Published:** 2016-02-24

**Authors:** Lorcan Kenny, Elisabeth Hill, Antonia F. de C. Hamilton

**Affiliations:** ^1^Centre for Research in Autism and Education (CRAE), University College London, Institute of EducationLondon, UK; ^2^School of Psychology, The University of NottinghamNottingham, UK; ^3^Department of Psychology, Goldsmiths, University of LondonLondon, UK; ^4^Institute of Cognitive Neuroscience, University College LondonLondon, UK

**Keywords:** social cognition, motor skill, theory of mind, imitation, action understanding

## Abstract

There is increased interest in the relationship between motor skills and social skills in child development, with evidence that the mechanisms underlying these behaviors may be linked. We took a cognitive approach to this problem, and examined the relationship between four specific cognitive domains: theory of mind, motor skill, action understanding, and imitation. Neuroimaging and adult research suggest that action understanding and imitation are closely linked, but are somewhat independent of theory of mind and low-level motor control. Here, we test if a similar pattern is shown in child development. A sample of 101 primary school aged children with a wide ability range completed tests of IQ (Raven’s matrices), theory of mind, motor skill, action understanding, and imitation. Parents reported on their children’s social, motor and attention performance as well as developmental concerns. The results showed that action understanding and imitation correlate, with the latter having a weak link to motor control. Theory of mind was independent of the other tasks. These results imply that independent cognitive processes for social interaction (theory of mind) and for motor control can be identified in primary school age children, and challenge approaches that link all these domains together.

## Introduction

Cognitive psychologists have traditionally studied human development within distinct domains. For example, social cognition (often exemplified by theory of mind tasks) has been studied separately from motor skill or visual skill. However, it is increasingly recognized that there may be links in the brain and cognitive systems underlying these different types of skill. The present paper aimed to examine the claim that motor and social skills develop in concert. To do this, we tested a large sample of primary school age children on a number of cognitive tasks designed to target theory of mind, mirror neuron systems, imitation and motor systems, and examined correlations between performance in these different domains.

### Mechanisms Underlying Motor and Social Behavior

In the present paper, we take a cognitive approach to development, meaning that we are interested primarily in the information processing mechanisms underlying different behaviors. We consider the information processing mechanisms of motor behavior, social behavior and all other behaviors including affect, language and perception, to all fall within the realm of cognitive neuroscience (Gazzaniga, 2004). We distinguish specific domains within this realm, and are guided in this classification by our knowledge of adult neuroscience. The present paper focuses on four distinct domains: social cognition, motor cognition, imitation and mirror neuron systems, which contribute in different ways to both motor behavior and social behavior.

Tasks used to assess social cognition in children include mentalizing (thinking about others’ thoughts), emotion and face recognition, and many other aspects of social behavior. The current study focuses on mentalizing in order to examine specific claims about the relationship between mentalizing and mirror neuron systems (Gallese et al., 2009; Hamilton, 2009). Tasks commonly used to assess motor cognition include performing hand actions, sequencing actions, whole body movements and balance. The current study focuses on planning and sequencing of hand actions, again because these are most closely linked to mirror neuron systems (Tunik et al., 2007). Tasks used to assess mirror neuron systems, which may contribute to both motor and social behavior, include imitation tasks and action understanding tasks. Developmentally, rapid improvements are seen in all of these tasks over infancy with development continuing in the primary school years (Bushnell and Boudreau, 1993; Jones, 2009).

### Hypothesized Links between Cognitive Domains

We can distinguish three different hypotheses for the relationship between different cognitive domains: (1) independent domains, (2) a single domain, and (3) domains interacting over development, and we describe each hypothesis in turn. Other hypotheses such as dynamical systems are also possible, but we return to these in the discussion (McClelland, 2010).

#### The Independent Domains Hypothesis

Traditional neurocognitive approaches tend to view different domains as independent. For example, different systems such as language, mentalizing, and motor control were considered largely distinct. In particular, mentalizing is considered as a highly specialized skill drawing on abstract abilities such as meta-representation (Perner, 1991) and quite unlike motor skills. If this were the case, we would expect development of mentalizing to be independent of development in motor control. Neuroimaging data also suggest that the different social and motor tasks described above draw on distinct brain networks. Mentalizing tasks reliably activate a brain network including medial prefrontal cortex and temporoparietal junction (Frith and Frith, 2003). Tasks involving understanding of actions or imitation typically activate a different brain network in the inferior parietal and inferior frontal lobe (Caspers et al., 2010). These brain regions are commonly referred to as the mirror neuron system (Rizzolatti and Craighero, 2004) and thus we will describe the tasks that activate these areas as mirroring tasks. Finally, motor tasks may engage mirror neuron systems but also draw on cerebellum and basal ganglia (Middleton and Strick, 2000). Thus, the domains of mentalizing, mirroring and motor control are at least partially distinct in terms of brain systems. The present paper aims to test if they are also distinct in terms of development.

#### The Single Domain Hypothesis

Even if brain and cognitive systems for mirroring and mentalizing are distinct in adulthood, it is possible that they develop from a single, primary system. For example, it has recently been suggested that ‘action cognition’ provides a basis for many different social-cognitive skills (Gallese et al., 2009). Building on the discovery of mirror neurons, which respond when a monkey performs an action and also sees another person act, it has been suggested that performing and understanding action is the developmental origin of human social skills. The idea put forth by the action cognition theory is that proficiency in social interactions fundamentally relies on the motor system to decode the movements of others to allow for attributions of intentions and mental states. The logic being employed here is this; when we observe another person’s movement our own motor system is activated in a way analogous to if we were performing the same action ourselves. It is this activation that allows us to introspect on what our intentions would be if we were performing that action and this would allow an inference about why the person is performing the action. Some papers have made further claims linking the mirror neuron system to empathy, theory of mind and social skills more broadly (Gallese et al., 2004) including the failure of social skill in autism (Rizzolatti et al., 2009). Under such a framework, adequate development of motor and mirror systems (jointly) is essential for the development of social skill, and there is a direct causal relationship between the development of these cognitive domains.

#### The Interactive Environment Hypothesis

A third hypothesis concerning the relationship between cognitive domains in development is an environmentally mediated hypothesis. This model sees the child’s development as a result of the interaction between the child and the environment, where changes to the environment can have a substantial effect on development. Achievements in one domain could thus have an impact on another domain via the environment. There is growing evidence for such cross-domain interactions at various developmental stages. For example, when a baby learns to sit up, she can see the world differently and adults may address her differently. This change in the environment may then lead to advances in the infant’s social skills, compared to her peers who are not yet able to sit. Evidence for this type of interaction can be seen in the finding that babies who have not yet learned to sit independently and those who have mastered the skill are comparable on measures of face processing, while those who are novice sitters perform worse, indicative of a reorganization taking place within the face processing system (Cashon et al., 2013). Another study finds that a baby who is given more opportunities to actively engage with objects shows an increase in orienting to faces relative to a baby given only passive experience with non-social objects (Libertus and Needham, 2011). Furthermore, crawling and walking in infants leads to changes in social interaction from parents (Campos et al., 2000; Karasik et al., 2011) and improvements in language skills (Iverson, 2010). Overall, the interactionist viewpoint predicts that different cognitive domains may be linked, but the underlying mechanism is external to the child. Such links may thus be weaker than a directly shared brain mechanism, or might only be measurable in longitudinal studies that track the child and her environment over time.

### Previous Studies of Social and Motor Development

As this brief review summarizes, the domains of mentalizing, mirroring, imitation and motor cognition could be unlinked, directly linked or linked via the environment. There are few previous studies of the development of motor and social skills in typical children. One large project tested 390 primary school children on fine and gross motor skills, theory of mind, emotion processing, and cognitive control. They found the motor skills correlated highly with IQ, language, social, and attentional skills. Parent ratings of social behavior were related to measured social skills but not motor skills (Dyck et al., 2004). Several studies have examined the relationship between motor and intellectual (not social) skills in children. For example, scores from standardized measures of gross cognitive and gross motor abilities are moderately and significantly correlated (Davis et al., 2011). Further, this study found that this relationship is largely accounted for by variances in visual processing and fine manual control, suggesting that these domains may well be linked via the environment. A number of studies have not found any reliable relationship between tasks tapping motor and social development, or links that are mediated by other higher order cognitive abilities such as memory and visual processing (Wassenberg et al., 2005; van der Fels et al., 2014).

Many more studies have examined motor and social skills in children with developmental disorders including autism and developmental coordination disorder (DCD). Children with autism are diagnosed on the basis of poor social skills, but up to 80% of them also have DCD (Green et al., 2009). Infants at risk of autism (due to having an older sibling with a diagnosis) are reported to have poor postural control (Flanagan et al., 2012) and difficulties with fine motor and grasping skills (Libertus et al., 2014). Motor difficulties such as these have been found to relate to later social and communicative ability (Bhat et al., 2012; Leonard et al., 2014) but although motor impairments are related to social abilities in autistic children, the relationship does not stand in their unaffected siblings (Hilton et al., 2012). In another study, autism severity as measured by scores on the Autism Diagnostic Observation Scale (ADOS), correlated with a measure of praxis that includes imitation tests, but not with more basic motor skills (Dziuk et al., 2007). Similarly, children with DCD differ from their typical peers in their use of social play (Kennedy-Behr et al., 2011). Additionally, autistic children’s scores on motor control assessments such as the Movement Assessment Battery for Children (M-ABC) or the Bruininks-Oseretsky Test of Motor Proficiency, Second Edition (BOT-2) predict the social behavior reported by parents (Hilton et al., 2007). A comparison between a group of autistic children and control groups matched for chronological age, motor skill, and developmental age suggests the impairment in motor skill is greater than would be expected based on ability alone (Staples and Reid, 2010). Similarly, a prospective study of children with DCD found that children with higher levels of motor clumsiness at age 5 had fewer social pastimes at age 15 (Cantell et al., 1994). These studies all suggest links between motor and social abilities in developmental disorders. However, many of these studies did not test specific components of motor and social cognition, relying instead on reports of behavior from parents or observations.

When more detailed cognitive assessments are carried out, results seem more mixed. In one intriguing study, children who were better able to adapt to lifting a heavy object also performed better on theory of mind tasks (Sabbagh et al., 2010), an effect that was not explained by age or executive function. Tests of motor cognition in autism suggest poor motor planning (Hughes, 1996) and posture knowledge (Dowell et al., 2009) in some cases but not others (Hamilton et al., 2007; van Swieten et al., 2010). Some studies report difficulties in chaining actions together in sequences (Cattaneo et al., 2007) but others do not (Pascolo and Cattarinussi, 2012). Detailed testing of visuomotor adaptation in children with autism did not find group differences (Mostofsky et al., 2004; Gidley Larson et al., 2008).

Similar variability is found in studies of how children with autism understand other people’s actions – a social component of motor cognition. Some studies report difficulties in answering questions about why a person did an action (Boria et al., 2009) or in predicting what will come next in a movie (Zalla et al., 2010) but other studies find no differences in the ability to make sense of hand gestures (Hamilton et al., 2007). Studies of imitation show intact performance on emulation tasks (copying the goal of an action) but poor performance on mimicry tasks (copying precise kinematic features; Hamilton, 2008; Edwards, 2014). For example, when participants with autism performed a motor task on a touch-screen computer that allowed careful matching of the motor, attentional and memory demands between the conditions, they still had poorer accuracy in the imitation condition compared to the emulation condition (Stewart et al., 2013). There is also a debate about how much imitation difficulties in autism relate to a motor or a cognitive deficit (Vanvuchelen et al., 2007). Overall, there is no single aspect of motor cognition that can be directly linked to poor social cognition – more research is needed to understand how motor and social developmental processes mesh together.

The present paper aims to measure motor abilities and social abilities in a large sample of children, using well-defined cognitive tasks. We aim to go beyond assessments of a child’s everyday behavior as measured in parent report or clinical measures. By tracking specific cognitive processes, we will be able to make much stronger links between the development of motor and social skills, and the neurocognitive theories of their origins. The present study uses a cross-sectional design, and thus cannot provide a causal account of how strengths in one domain might contribute to strengths in a different domain. However, it can provide an initial measure of the strength of inter-domain links, with a view to future longitudinal studies. In the following section, we set out and justify the tasks used in the present study.

### Testing Cognitive Development

To test for links between the motor, mirror and mentalizing domains, we needed a set of cognitive tasks that could measure children’s performance in each area. For the mentalizing domain, we used theory of mind tasks, which have been well-studied over the last 30 years. Performance on explicit tests of theory of mind becomes reliable from about age 4 (for review see, Wellman et al., 2001). Using a variety of tests with differing complexity, developmental improvements can be traced from the age of 3 years up to 8 years or even into adolescence (Dumontheil et al., 2010; Calero et al., 2013). In the present paper, we used a battery of theory of mind tasks drawn from past work (Wellman and Liu, 2004) as our measure of mentalizing ability.

To measure the intersection of social and motor processes, we used two types of task which, in adults, engage mirror neuron systems in the brain, namely action understanding tasks and imitation tasks (Buccino et al., 2004; Iacoboni et al., 2005). To assess action understanding, we used a gesture recognition task derived from studies of patients with apraxia (Mozaz et al., 2002) in which a child must choose which photograph of a hand gesture would best fill the gap in a cartoon action. We also used a grasp-intention task in which a child must use a photograph of how an object is grasped to decide ‘why’ the actor is holding the object –to move it or to use it (Boria et al., 2009). To assess imitation abilities, we instructed children to imitate a series of hand/arm actions and measured accuracy. Instructed imitation is likely to be a better measure of mirror system function than the propensity to spontaneously imitate (Vivanti, 2015).

Cognitive tests of motor systems are also not easy to find. Studies have traditionally focused on the performance of tasks relevant to daily life, such as walking or writing (Sugden, 2007). Here we aimed to retain a cognitive focus and use tasks that can be linked to specific motor processes, including motor planning, sequencing and prediction. Thus, we used a bar task which requires the child to consider the end posture in an action sequence before beginning to move – a measure of motor planning (Cohen and Rosenbaum, 2004; Rosenbaum et al., 2006). Motor planning skills improve over 3–10 years-old age range (Stöckel et al., 2012; Weigelt and Schack, 2015). We also used a sequencing task (Harrington and Haaland, 1992) which assessed how long it took to switch between different actions rather than performing the same action repeatedly.

### The Present Study

The present study aimed to measure specific cognitive processes underlying motor and social skill in primary school age children, and to determine how they develop together. We used several tasks to measures performance in four different cognitive domains – of theory of mind, action understanding, imitation and motor control, as detailed above. The present paper focuses only on the domain-level of analysis because the theories that motivated this study are specified at that level. Analysis of performance on individual tasks within each domain will be presented in a different paper. We tested a large sample of children (*n* = 101) to obtain good statistical power. A power analysis shows that obtaining a medium effect size with 95% power in a multiple regression with seven predictors requires a sample size of 89 participants. If motor and cognitive skills develop from distinct cognitive systems, then performance on the theory of mind tasks will not be related to performance on the mirroring or motor tasks. In contrast, if the engagement of a single cognitive system (such as the mirror neuron system) drives both motor and social development, then the different cognitive domains will correlate tightly across participants. If motor and social skills are linked only via environmental effects, then weak correlations between domains may be observed as well as with IQ.

## Materials and Methods

### Participants

We invited children aged between 4 and 12 years-old to participate in the project. Families were contacted through local primary schools and a database of people interested in research. All parents completed an informed consent form before their children took part, and the study was approved by the University of Nottingham School of Psychology ethics board.

For the first phase of the project, parents of 188 children completed four questionnaires – the Developmental Coordination Disorder Questionnaire (DCDQ; Wilson and Crawford, 2010), The Social Responsiveness Scale (SRS; Constantino and Gruber, 2005) and the Conners 3 ADHD index (Conners 3AI; Conners, 2008), as well as a family background questionnaire collecting data on child’s age, languages spoken, socioeconomic status (based on parents’ jobs) and any developmental concerns about their child. A more detailed analysis of this phase of the project will be reported elsewhere.

Of the 188 children, 101 participated in the second phase of this project which involved detailed cognitive testing. Data from all 101 is reported here. This sample was not selected entirely at random. First, the availability of children and schools for testing constrained the choice of participants. Second, children whose scores on either the SRS or the DCDQ were toward either end of the distribution of scores obtained from the phase one sample were deliberately oversampled. This is because a fully random sample would include many children with mid-range scores. By oversampling children with extreme scores, we maximized the variance in abilities among the children tested and increased our power to detect associations between the different measures in our study. None of the children tested had a formal diagnosis of developmental delay, but some were receiving additional support from their school or undergoing assessments for difficulties.

### Cognitive Testing

The 101 children who completed cognitive testing were assessed by a trained researcher in a quiet room at their school or at the University of Nottingham. They completed the following tasks spread over 2–4 sessions.

### Mentalizing Assessment

This included widely used theory of mind tasks – the diverse desires task, diverse beliefs task, knowledge access task, explicit false-belief task, implicit false-belief task, and contents false-belief task were used as in Hamilton et al. (2007). A child was given 1 point for each task where they passed control questions and demonstrated theory of mind. Children completed six sequences of a picture-sequencing task (Baron-Cohen et al., 1986) and were given 1 point for each fully correct answer and a score of 0.5 was given if the final picture of a sequence was correct but the second and third were in the wrong order. Children completed six trials of a penny-hiding task (Gratch, 1964) which is an interactive measure of strategic mentalizing. The child was given one point for each appropriate attempt to hide a coin from the experimenter. Scores for all the theory of mind tasks (six classic tasks, six picture sequencing trials, and six penny hiding trials) were totaled for each child. The data were then linearly scaled so that the sample mean was 0 and standard deviation was 1. Inspection of the quantile plots in R showed no substantial deviation from normality so no further data transformations were applied.

### Mirror System Assessment

This included tests of imitation, intention understanding and posture knowledge. In the imitation task, the experimenter sat opposite the child and asked the child to watch the action and then to copy as closely as possible as if looking in a mirror. The experimenter demonstrated with the hand mirroring the child’s dominant hand, and the child used his/her dominant hand to respond. One practice trial was given to ensure the instructions were understood. Children performed six trials with meaningful actions and six with meaningless actions (blocked, with block order counterbalanced) and performance was scored from video. Two trained raters coded all videos for overall imitation quality (0, 1, or 2) and specific error types, but only the former are reported here. Reasonable inter-rater reliability was achieved (Cohen’s weighted kappa was 0.75). Quality scores were summed for each child and averaged across raters, giving a score out of 24. As before, data were linearly scaled to have a mean of 0 and standard deviation of 1. Inspection of the quantile plots in R showed deviation from normality that was best corrected with by squaring the values, so this transformation was applied.

The intention understanding task was based on Boria et al. (2009). New picture stimuli were generated showing a hand touching, lifting or using a variety of everyday objects. Stimuli were piloted with typical adults to ensure that the objects and actions could be clearly identified. On each trial, the child first saw a card with a picture of an everyday object and was asked – what is it? Responses were 99.7% correct. Then the child was asked if the hand was holding or touching the object. For the holding images, the child was asked – why is he holding it? To use it or to move it? 10 different objects were photographed, resulting in 10 hold-to-use photos and 10 hold-to-move photos. Responses to the ‘why’ question for each of these 20 photos were scored with 1 point for each correct answer, giving a score out of 20. The posture knowledge task was identical to that used by Hamilton et al. (2007). On each trial, the children saw a cartoon of a person performing an action with the hands missing, together with three photos of hands in different postures and were asked ‘which hands fill the gap?’ Correct responses were given 1 point with a total score out of 16. Scores on the intention understanding task and the posture knowledge task were summed for each child. As before, data were linearly scaled to have a mean of 0 and standard deviation of 1. Inspection of the quantile plots in R showed no substantial deviation from normality so no further data transformation was applied.

### Motor Assessment

This included two tasks – a test of motor planning and a test of motor sequencing. The motor planning task was based on Rosenbaum et al. (1990), and previously used in autism research (Hughes, 1996; Hamilton et al., 2007). On each trial, the child saw a bar with two ends of different colors resting horizontally on a rest 10 cm above the table, and two targets (paper disks on the table) of different colors. They were asked to place one end of the bar on one of the targets (e.g., place the red end on the black target). On four trials, this could be comfortably achieved by grasping the bar at the start of the trial with an overhand grip, while on four trials the less-common underhand grip was more appropriate. Typical adults are able to plan their movements to end in a comfortable posture by adopting a less-common posture at the start of the action (Rosenbaum et al., 2006) and this ability develops over childhood (Adalbjornsson et al., 2008). Thus, this task assesses motor planning. Children received a score out of 8 with one point for each trial where the appropriate grip was used. Motor planning scores were linearly scaled to have a mean of 0 and standard deviation of 1.

The motor sequencing task was based on Harrington and Haaland (1992), and aimed to assess motor speed and the ability to switch between actions. The apparatus was a set of black boxes each with one movable part – a switch to flick, a button to push or a dial to twist. On each trial, the experimenter prepared an array of five of these boxes in a specific order (e.g., flick, twist, twist, flick, flick). When the child was ready with his/her hand on the start location on the left of the desk, the experimenter revealed the array of boxes and the child moved his/her hand along the array performing each action in turn. Trials were videoed and the time from moving away from the start-location to moving away from the last action was coded. 40% of videos were second scored and the correlation between the two scorers was *r* = 0.93. Some box sequences contained no transitions (e.g., push-push-push-push) while others contained one, two, three or four transitions (e.g., push-twist-push-twist-push). Data were analyzed by fitting each child’s movement time on each trial to a linear model with five predictors: one for each action (flick/push/twist), one for transition time (coded 1 for switch and 0 for a stay) and one for learning (a linear decrease over the 15 trials). Outliers in these parameter estimates were identified as values 3 standard deviation above/below the mean (*n* = 8 out of 404 data points) and replaced with the group mean.

To combine the motor task scores into a single score for each child, the following transformations were applied. First, values for each score (motor planning; flick-time; push-time; twist-time; transition time; learning) were linearly scaled so that each full set of scores had a mean of 0 and standard deviation of 1. The combined motor score was then defined as: – (*flick-time + push-time + twist-time)/3 + motor planning – transition time* using the linearly scaled scores for each. Timing values were negative to ensure that larger values reflect better performance, consistent with other data in this analysis. Inspection of quantile plots showed no substantial deviation from normality so no further data transformation was applied.

### IQ Assessment

Raven’s colored progressive matrices (Raven et al., 1998) were used to measure each child’s non-verbal IQ (nvIQ). Raw scores (not normed scored) were then linearly scaled to have a mean of 0 and standard deviation of 1, in line with other data in this analysis. Inspection of the quantile plots in R showed no substantial deviation from normality so no further data transformation was applied.

### Parent Report Scores

Parents completed the SRS, the DCDQ and Conners 3 AI scale. Scores on these scales correlated highly, and a detailed analysis of these data will be reported elsewhere. Descriptive statistics on the raw scores are presented in **Table 1** to illustrate the sample of children tested here. The present study focused on cognitive performance, so we combined the parent report scores into a single factor reflecting parent concerns. To create the factor, we first inspected the raw scores on each of the three parent-report instruments (SRS, DCDQ and Conners) using quantile plots in R. DCD-Q scores were then squared to reduce the deviation from normality. SRS scores and Conners scores were inverted so that a larger value indicates better performance (to be consistent with all other measures). Each transformed score was then linearly scaled to have a mean of 0 and standard deviation of 1, and the scores were summed for each child. This gives a combined parent-report measure that weights social, motor and attentional concerns equally, and which gives higher values to children showing better performance across these domains. Each child’s primary caregiver was asked for their current occupation and responses were coded using the International Standard Classification of Occupations (ILO, 2012) where higher values indicate lower socioeconomic status.

**Table 1:** Characteristics of 101 participants.

	Mean	*SD*	Minimum		Maximum
Age (years)	7.88	1.69	4.88		11.55
SES	3.13	1.5	1		9
Attention (Conners)	5.4	6.0	0		20
Motor skill (DCDQ)	56.6	14.2	16		75
Social development (SRS)	40.9	31.8	0		145
nvIQ (Raven’s raw score)	25.4	6.6	11		36
				
Handedness	10 left	3 ambidextrous		88 right	
Gender	60 male	41 female			
Any parental concern about possible developmental issues	76 no	35 yes			


### Statistical Analysis

Data for 101 children were available. As described above, scores on each individual task were transformed to ensure that the data were normally distributed and linearly scaled to ensure that higher values reflect better performance. This gave summary scores for each of the following domains: theory of mind; imitation; mirroring; motor skill; non-verbal IQ; parent report; together with age and gender data for each child. The correlations between each of these sets of summary scores were calculated. Then four general linear models (GLMs) were set up to test which factors predicted each of the four cognitive domains of interest. For example, the Theory of Mind model tested how their imitation score, mirroring score, motor score, nvIQ, parent score, age and gender, predicted a child’s Theory of Mind score. The imitation model tested how their ToM score, mirroring score, motor score, nvIQ, parent score, age and gender, predicted a child’s imitation score. Effectively, these models tested whether performance in each cognitive domain was accounted for by general effects (e.g., nvIQ) or if performance was closely linked to another cognitive domain.

To further probe the data, we conducted a number of exploratory analyses. First, we excluded all children for whom parents had indicated a developmental concern, that is, all children who are receiving additional help at school or undergoing assessments for a developmental disorder. Then we re-ran the GLM models on the remaining sample of typical children. This checks if our results are driven only by the atypical children in the sample. Second, we split the sample into 3 age bands with equal numbers of children in each band. We then re-ran the GLM models on these three samples. This checks if links between different domains might be apparent in only some age ranges. However, both these analyses are conducted on smaller samples and have reduced statistical power.

To explore cross-domain links in the full sample without confounds of age, we examined correlations between the residuals of each domain after removing effects of age, non-verbal IQ and gender. Specifically, we set up a GLM predicting theory of mind performance as a function of age, non-verbal IQ and gender. We took the residuals from this model as a measure of each child’s theory of mind performance after age, gender and IQ effects are removed. In the same way, we set up three separate GLMs of mirroring performance; motor performance and imitation each as functions of age, non-verbal IQ and gender. We took the residuals of all four models and examine the pattern of correlations between them. This gives insight into the relationship between different cognitive domains across the full sample of 101 children but without any confounding effects of age or IQ.

## Results

### Correlations

**FIGURE 1:**
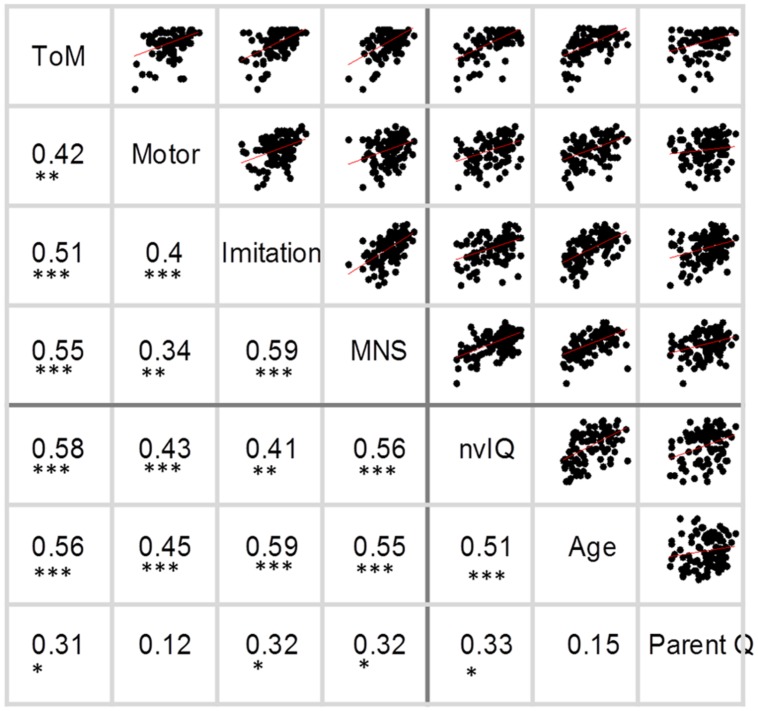
**Correlations between all variables.** In the upper triangle, each dot represents one participant and the line-of-best-fit is shown in red. In the lower triangle, values are Pearson’s *r*. ^∗^ indicates correlations meeting the *p <* 0.002. Bonferroni corrected threshold. ^∗∗^ indicates correlations at *p <* 0.00001. ^∗∗∗^ indicates correlations at *p <* 0.0000001.

The correlations between all the scores in the complete dataset are illustrated in **Figure 1.** Note that correction for multiple comparisons has not been applied, but an appropriate Bonferroni threshold for 21 comparisons would be *p* < 0.002. Correlations between almost all measures were high; with the exception that parent questionnaire scores did not correlate with motor scores, theory of mind scores or age, using the corrected significance threshold.

### General Linear Models

Four GLM analyses were performed, to test the relationship between performance on the cognitive tasks and parent report in different domains. Results of these analyses are presented in **Table 2.** Model 1 found that theory of mind scores could be predicted based on age and non-verbal IQ but were not related to motor, imitation or mirror system performance. Model 2 found that motor scores could be predicted from gender and non-verbal IQ, with imitation skill as a marginal predictor. Note that this model had a weaker overall fit (adjusted *R*^2^ = 0.28) than any of the other models. Model 3 found that mirror system scores could be predicted from imitation scores and non-verbal IQ, but motor and theory of mind scores did not contribute. Model 4 showed that imitation scores could be predicted from age, parent questionnaires and mirror system scores, with motor scores as a marginal predictor.

**Table 2:** Results of the GLM analyses performed to test the relationship between performance on the cognitive tasks and parent report measures.

	*b*	*SE*	*t*	ß	*p*
**Model 1: Theory of Mind**					
Overall model	*F* = 12.69, df = 7,93, *p* < 0.0001, adj *r*^2^ = 0.45				
Intercept	-0.96	0.50	-1.92	-0.96	0.058
Gender	-0.09	0.16	-0.54	-0.09	0.592
Age	0.13	0.06	2.09	0.13	**0.039^∗^**
Parent Questionnaires	0.03	0.03	0.99	0.03	0.325
Motor	0.05	0.05	0.94	0.05	0.350
Imitation	0.12	0.11	1.19	0.13	0.237
MNS	0.10	0.07	1.50	0.10	0.137
Non-verbal IQ	0.26	0.10	2.63	0.26	**0.010^∗^**
**Model 2: Motor**					
Overall model	*F* = 6.53, df = 7,93, *p* < 0.0001, adj *r*^2^ = 0.28				
Intercept	-1.01	1.06	-0.95	-1.01	0.343
Gender	-0.79	0.32	-2.48	-0.79	**0.015^∗^**
Age	0.19	0.13	1.45	0.19	0.150
Parent Questionnaires	-0.07	0.07	-1.01	-0.07	0.313
ToM	0.20	0.22	0.94	0.20	0.350
Imitation	0.37	0.22	1.72	0.37	0.089
MNS	-0.06	0.14	-0.41	-0.06	0.682
Non-verbal IQ	0.41	0.21	1.99	0.42	**0.049^∗^**
**Model 3: Mirroring**					
Overall model	*F* = 13.76, df = 7,93, *p* < 0.0001, adj *r*^2^ = 0.47				
Intercept	-1.21	0.79	-1.52	-1.21	0.132
Gender	0.01	0.25	0.05	0.01	0.961
Age	0.15	0.10	1.56	0.15	0.122
Parent Questionnaires	0.05	0.05	0.94	0.05	0.352
ToM	0.24	0.16	1.50	0.24	0.137
Motor	-0.03	0.08	-0.41	-0.03	0.682
Imitation	0.48	0.16	3.02	0.48	**0.003^∗∗^**
Non-verbal IQ	0.41	0.16	2.62	0.41	**0.010^∗^**
**Model 4: Imitation**					
Overall model	*F* = 13.49, df = 7,93, *p* < 0.0001, adj *r*^2^ = 0.47				
Intercept	-1.67	0.47	-3.56	-1.67	0.001
Gender	0.24	0.15	1.55	0.24	0.125
Age	0.19	0.06	3.34	0.19	**0.001^∗∗^**
Parent Questionnaires	0.07	0.03	2.18	0.07	**0.032^∗^**
ToM	0.12	0.10	1.19	0.12	0.237
Motor	0.08	0.05	1.72	0.08	0.089
MNS	0.19	0.06	3.02	0.19	**0.003^∗∗^**
Non-verbal IQ	-0.10	0.10	-1.00	-0.10	0.318


*^∗^*p* < 0.05, ^∗∗^*p* < 0.01.*

**FIGURE 2:**
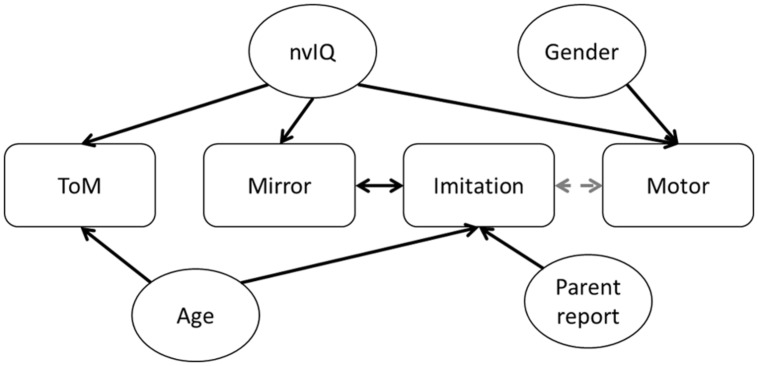
**Summary of significant effects found.** The four cognitive domains are listed across the centre. Each solid arrow indicates a factor which predicted performance in that cognitive domain. Each dashed arrow indicates a marginally significant predictor.

Overall, the correlation analysis and the GLM models provide a consistent picture. Imitation and mirror system performance are related to each other, and are weakly linked to motor skill. Theory of mind scores are linked to nvIQ but not to any of the motor scores. To summarize these results, we illustrate the factors which reliably predict performance in each of the four cognitive domains in **Figure 2.**

### Further Exploratory Analyses

We performed several exploratory analyses to check the robustness of our results. First, we implemented the four GLM models on the data from the 76 children for whom there was no parental report of any developmental concerns. Results for the ToM model showed that parent report scores were a reliable predictor of performance (*p* = 0.039) but no other predictors were significant. Results for the Motor model showed that gender (*p* = 0.034) and nvIQ (*p* = 0.026) were reliable predictors, replicating the pattern found in the full sample. Results for the mirroring model showed that imitation (*p* = 0.0049) and nvIQ (*p* = 0.027) were reliable predictors, replicating the pattern found in the full sample. Results for the Imitation model showed that age (*p* = 0.034), parent reports (*p* = 0.028) and Mirroring (*p* = 0.0049) were reliable predictors, replicating the pattern found in the full sample. Thus, the analysis of data from only children with no developmental concerns gave a very similar pattern to the full data sample, with no indication of stronger relationships between cognitive domains in this more homogenous sample.

Second, we split the data into three sub-samples by age: a young group of 33 children aged 4.8–6.7 years; a mid-aged group of 33 children aged 6.8–9 years and an old group of 34 children aged 9–11.5 years. We implemented the GLM models on data from each sub-sample separately. In these 12 GLMs, the only predictors meeting the *p* < 0.05 threshold were: in the young group, nvIQ predicts mirroring performance; in the mid-aged group, Mirroring, Imitation, and Motor performance were all reliable predictors of each other; in the old group, Ravens predicted motor performance and age predicted mirroring performance. There were no indications of strong relationships between the specific cognitive domains that differ from our main report in these subsamples, though we note that these analyses are likely to be underpowered.

Third, we aimed to examine each cognitive domain without confounding effects of age and IQ. To do this, we modeled performance in each of the four cognitive domains separately as a function of age, non-verbal IQ, and gender. We took the residuals from each model as measures of each child’s domain performance without any age, gender or IQ effects. We took the residuals of all four models and examined the pattern of correlations between them. This gives insight into the relationship between different cognitive domains across the full sample of 101 children but without any confounding effects of age or IQ. Correlations in this model were given in **Table 3.** The only correlation which survives an appropriate correction for multiple comparisons (*p* < 0.005) is the correlation between imitation and mirroring, a result also found in our primary analysis.

**Table 3:** Results of the residuals correlation.

	ToM	Motor	Imitation	MNS	Parent report
ToM		0.12	0.23	0.23	0.16
Motor	0.248		0.17	0.03	-0.04
Imitation	0.019	0.089		0.36	0.26
MNS	0.020	0.802	0.000		0.19
Parent report	0.117	0.661	0.009	0.054	


*Upper triangle indicates correlation coefficient, lower triangle indicates *p*-values.*

## Discussion

In this study of 101 children, we examined cognitive performance across the motor and social domains. We found that performance on theory of mind tasks was independent of action understanding, imitation, and motor skill. However, action understanding and imitation were closely related, and somewhat linked to motor skill. These results have important implications for theories of how different cognitive domains develop and are related to one another.

In the introduction, we set out three possible models for the relationship between social and motor skills. These skills could develop independently, they could be fully integrated or they could be linked via the environment. The present data do not give support to a wholly integrated model (hypothesis 2) such as the action cognition framework set out by Gallese et al. (2009). With that theory comes the testable hypothesis that performance on tasks tapping motor cognition, the mirror neuron system and social cognition will all be necessarily related. In our data, imitation and action understanding were closely linked, and weakly correlated to motor cognition. This supports the claim that the process of understanding another agent’s action involves the recruitment of a perception-action network. However, the social abilities measured with theory of mind tasks were independent of mirror and motor skills. This argues against the hypothesis that difficulties in social cognition cascade downstream from impaired motor cognition, or that shared understanding of perception and action contributes to mentalizing. It remains possible that social and motor cognition could be more integrated at earlier stages of development than was considered by the present study and it may be that it becomes increasingly modularized across development or the relationship may differ when different components of motor cognition are considered (e.g., Sabbagh et al., 2010).

In contrast, our data align well with the cognitive task distinctions suggested by adult neuroimaging data and by traditional cognitive theories. In neuroimaging studies, action understanding and imitation engage the same brain systems; partially overlapping with other motor skills, while theory of mind engages different systems. Similarly, in our developmental data, action understanding and imitation are mutually predictive, and have a weak relationship to motor skill.

Our data cannot rule out the possibility that different cognitive domains interact over developmental time, linked by the environment. There is evidence for this in some longitudinal studies. For example, questionnaire data from over 62,000 children as part of a cohort study revealed that motor skill at 18 months predicted communication skills at 3 years (Wang et al., 2014). Bart et al. (2007) found that motor skills in kindergarten predicted study skills and disruptive behavior (but barely predicted social behavior) a year later. Ommundsen et al. (2010) found that motor skill in 1st grade predicted social status in 4th grade, measured in 80 children. Note that all these studies used self-report or teacher report measures of social behavior, rather than cognitive tests. Thus, it remains unclear if motor cognition can be directly linked to social cognition in a longitudinal fashion.

Our data also cannot rule out the possibility that there are links between performance on specific tasks within different cognitive domains, which does not emerge when performance in each domain is combined as we have done here. For example, Davis et al. (2011) found that subscores in tests of visual processing and fine manual control were correlated in a group of 4–11 years-old children and that this task-level effect drove the link between motor and intelligence domains. It is possible that there are similar relationships between specific tasks in our study, but unfortunately there are too many tasks and not enough participants to implement the PCA or task-level analysis used by Davis et al. (2011). It would be interesting to test if specific tasks or specific cognitive sub-components are linked across domains in future work.

### Clinical Relevance

The results of the present study have implications for how we understand disorders of both social and motor cognition. For instance, if motor and social skills develop independently of each other, as the data presented here suggest, then it is not clear why there is such a high degree of co-morbidity of autism and DCD. The present study did not test children with a diagnosis of autism or DCD, but some children were undergoing assessments for a variety of developmental concerns. This enabled us to test a larger and more variable sample. However, without participants diagnosed with disorders, it is not possible to know if the same relationships between motor and social skills hold on that sample. It is possible, for example, that motor cognition and theory of mind are closely linked in autism even if they are not linked in a typical sample. It is possible that the relationship is qualitatively different in atypical populations and that cognitive systems may be more interdependent and have increasing cascade effects on each other. Alternatively, it may be that an underlying neurological susceptibility to cognitive delay or deficit may similarly affect abilities that are reasonably unrelated in typical development.

The independence between mentalizing ability and motor cognition in this study has implications for the design of interventions for those who are at a social or motoric disadvantage. For example, there have been studies exploring the effects of interventions targeting imitation skill in autism to improve social emotional functioning (Ingersoll, 2012). While Ingersoll found improvements in social emotional functioning when children were followed-up were related to treatment it was not clear that improvements in imitation was the mechanism through which these improvements were manifest.

### Strengths and Weaknesses

This study is limited in some ways. Most of our experimental measures were based on previously published work, to ensure robustness. However, our measure of motor sequencing was novel and has not previously been used with children. The sequencing task requires children to complete a series of actions, where the number of switches from one action type to another can vary. Reaction time was measured from video coding which may also have introduced an element of error. Furthermore, the novelty of this task makes it difficult to determine what optimal performance should look like. Some of the measures used produced some ceiling effects and so were not capturing the full variance that exists in the population for these measures. This was particularly the case for the Theory of Mind tasks and despite normalizing the distribution it may be that the distribution of scores would have had greater variance in a younger sample or if more implicit measures of mentalizing were used. Furthermore, the questionnaires used to measure parental reports of a child’s behaviors are designed to be used as screening measures for differentiating children who potentially have a clinical diagnosis from those who do not and as such they were not designed to measure ability equally across the entire range of typical social, motor and attentional ability.

There are also several statistical and analysis issues which could affect our conclusions. First, our sampling strategy involved selecting children for cognitive testing who had extreme scores on the parent report measures, in order to maximize the variance in our sample. While none of the children in our sample have a clinically diagnosed developmental disorder, it is possible that this sampling method could bias our results if there are discontinuities between typical and atypical development. The fact that we find similar results when we analyze data only from children with no developmental concerns argues that our sampling method did not introduce strong biases into our analysis. Second, it is possible that performance in different cognitive domains changes non-linearly with age. Our analysis uses only linear models and cannot capture this. Substantially larger sample sizes would be needed to examine non-linear age effects. Finally, we tested children across a wide age range but did not have enough participants to break down the dataset into smaller, more homogenous groups to test if the relationship between cognitive domains changes over development.

This study did, however, have some areas of strength. First, the large sample offered good statistical power to detect relationships between multiple variables. The results we found are consistent in both our primary GLM analysis, which takes a conservative approach to testing for strong relationships between cognitive domains, and in three further exploratory analyses which tested for these relationships in sub-samples of the data. Second, the present study employed cognitive tasks that were measuring children’s abilities in certain domains rather than their parent’s perception of their ability relative to normative performance. This is a very important distinction as it allows for a more fine-grained exploration of the component aspects of cognition that would be too difficult to elicit in questionnaires. The relationship between parent measures in these domains with children’s performance on related tasks from the current sample will be explored in more detail elsewhere.

## Conclusion

The data presented in the current study suggest that different domains of social and motor skill, specifically the theory of mind domain and the mirroring domain, are relatively independent in this sample. This argues against a ‘single domain hypothesis,’ but is compatible with an ‘independent domains’ hypothesis or an ‘interactive environment’ hypothesis. Longitudinal data will be needed to discriminate and further test these hypotheses, and thus to better understand the ways in which different cognitive processes interact across motor and social development. This is especially the case in developmental disorders when the development of these cognitive capacities may be incommensurate with each other and in turn with the requirements of the environment, leading to functional impairment. Intervention studies should be used to not only address questions of efficacy and effectiveness at improving motor and social proficiency but also in order to test the mechanisms through which social and motor skill develop in concert or autonomously. We suggest that future research should adopt a cognitive approach to the measurement of motor skill, mirror neuron system functioning, and social cognition in clinical and non-clinical control groups in order to test and develop our understanding of the mechanisms of development.

## Author Contributions

AH, LK and EH designed the study. LK collected the data. AH & LK analysed the data. AH, LK and EH wrote the paper and have approved it for publication.

## Conflict of Interest Statement

The authors declare that the research was conducted in the absence of any commercial or financial relationships that could be construed as a potential conflict of interest.

The reviewer KL and handling Editor declared a current collaboration and the handling Editor states that the process nevertheless met the standards of a fair and objective review.
